# Characteristics and quality of clinical practice guidelines for depression in adults: a scoping review

**DOI:** 10.1186/s12888-019-2057-z

**Published:** 2019-02-20

**Authors:** Jessica Hanae Zafra-Tanaka, Sergio Goicochea-Lugo, David Villarreal-Zegarra, Alvaro Taype-Rondan

**Affiliations:** 1 Universidad Peruana Cayetano Heredia, CRONICAS Centre of Excellence for Chronic Diseases, Lima, Peru; 2 Independent researcher, Lima, Peru; 3 Instituto Peruano de Orientación Psicológica, Lima, Peru; 4 Universidad San Ignacio de Loyola, Unidad de Investigación para la Generación y Síntesis de Evidencias en Salud, Av. La Fontana 550, La Molina, Lima, Peru

**Keywords:** Major depression, Clinical practice guidelines, Quality, AGREE-II

## Abstract

**Background:**

Clinical Practice Guidelines (CPGs) should follow an adequate methodology using an evidence-based approach in order to provide reliable recommendations. However, little is known regarding the quality of CPGs for Depression, which precludes its adequate use by stakeholders and mental health professionals. Thus, the aim of this study was to conduct a scoping review to describe the characteristics and quality of CPGs for Depression in adults.

**Methods:**

We searched CPGs for Depression in adults in eighteen databases. We included those that were published in English or Spanish between January 2014 and May 2018 and were based on systematic reviews of the evidence. Two independent authors extracted the characteristics, type and number of recommendations, and quality (using the Appraisal of Guidelines for Research and Evaluation-II [AGREE-II]) of each included CPG.

**Results:**

We included eleven CPGs, of which 9/11 did not include the participation of patients in the development of the CPG, 4/11 CPGs had a score ≥ 70% in the overall evaluation of AGREE-II, and 3/11 CPGs had a score ≥ 70% in its third domain (rigor of development). In addition, only 5/11 CPGs shared their search strategy, while only 4/11 listed the selected studies they used to reach recommendations, and 7/11 CPGs did not clearly state which methodology they used to translate evidence into a recommendation.

**Conclusions:**

Most of evaluated CPGs did not take into account the patient’s viewpoints, achieved a low score in the rigor of development domain, and did not clearly state the process used to reach the recommendations. Stakeholders, CPCGs developers, and CPGs users should take this into account when choosing CPGs, and interpreting and putting into practice their issued recommendations.

**Electronic supplementary material:**

The online version of this article (10.1186/s12888-019-2057-z) contains supplementary material, which is available to authorized users.

## Background

Depression is recognized as an important public health issue. By the year 2015 it affected around 4.4% of the population [1], and by the year 2016 it was responsible for approximately 6.75% of years lived with disability in adults worldwide [2]. Different actions are needed to improve the care of people suffering from depression, such as the development and implementation of adequate clinical practice guidelines (CPGs).

CPGs are classically defined as a set of “systematically developed statements to assist practitioner and patient decisions about appropriate health care for specific clinical circumstances” [3]. CPGs can help to close gaps between evidence and policy, issuing recommendations in favor of the use of effective interventions and against the use of futile interventions [4]; CPGs are needed to establish reliable recommendations achieved through a clear methodology [5]. However, the methodology used for CPGs development is usually poorly defined and varies widely in content and quality between and within developing institutions [6], which may lead to inconsistency between recommendations. Accordingly, a study that compared recommendations from 23 CPGs published between 2007 and 2017 found high inconsistency in recommendations for second and third-line of pharmacological treatment of depression [7].

It is of great importance that stakeholders and mental health professionals are aware of the characteristics and quality of currently used CPGs for depression. The quality of the CPGs should be taken into account when interpreting and putting in practice recommendations issued in these CPGs. However, to our knowledge, only one study has assessed the quality of CPGs for depression and anxiety in children and youth [8], and we have not found studies that have described the quality of CPGs for depression in adults. Thus, the aim of this study was to describe the characteristics and quality of CPGs for depression in adults.

## Methods

We performed a scoping review of CPGs for depression in adults published in the last 5 years and evaluated characteristics regarding scope, methods used to reach recommendations, and grading the strength of recommendations. We also assessed the quality of each CPG. The PRISMA guidelines for scoping reviews (PRISMA-ScR) were used to secure adequate reporting and to guarantee the replicability of the study [9].

A scoping review is “a form of knowledge synthesis, which incorporate a range of study designs to comprehensively summarize and synthesize evidence with the aim of informing practice, programs, and policy and providing direction to future research priorities” [10]. It is similar to a systematic review, but it mainly differs on the objective they pursue. While a systematic review aims to find an answer to a well-defined question, a scoping review can be used identify, map and discuss certain characteristics in papers or studies [11]. Given that our aim was to identify CPG and their characteristics, we decided to use the later.

### Eligibility criteria

We included all CPGs, defined as a document that aimed to state recommendations, that fulfilled the following criteria: assessed screening, diagnosis or management of depression in adults; were published or totally/partially updated in the last 5 years (January 2014- May 2018); full-text were available in English or Spanish; and used systematic reviews of the evidence to guide their recommendations. We decided to include only CPGs based on systematic reviews, based on the current CPG definition which states that they should be designed based on a systematic review of the evidence [12].

We excluded those CPGs that assessed specific types of depression such as bipolar or psychotic depression, or specific types of populations such as depression in patients with cancer or in older people after a stroke.

### Search strategy

We performed a comprehensive search in eighteen databases. Our search strategy included terms related to depression and guidelines/practice guidelines. Searchers were performed by two independent researchers (JHZT and DVZ), and the last update was run in June 2018 (see Additional file 1).

### Study selection

Two independent researchers (JHZT and DVZ) evaluated if the CPGs met the eligibility criteria for inclusion. When there were discrepancies, a consensus was reached after debating them among all the authors.

### Data extraction

The following characteristics were extracted from the CPGs: authors; year of publication; country; involvement of patients or their representatives in the CPG development process; methodology used to reach recommendations; methodology used for grading the strength of recommendations; usage of minimally important difference (MID) when evaluating the effect of interventions; and the number of recommendations and good clinical practice (GCP).

We defined a recommendation as “all the statements in favor or against an intervention based on systematic reviews of the evidence, which typically include a formal assessment of the benefits and drawbacks of available treatment options” [13]. All the statements that synthetize opinions from an organized group of experts (expert consensus) and aim to describe “customary and expected care to be offered to patients” in situations where little to no evidence is available were considered as GCP [13].

We defined MID as a measure of the “smallest change in patient-reported outcomes of interest that patients perceive as important” [14].

### Quality appraisal

To assess the quality of CPGs we used the Appraisal of Guidelines Research and Evaluation II (AGREE-II), which has 23 items distributed in six domains (scope and purpose, stakeholder involvement, rigor of development, clarity and presentation, applicability, and editorial independence). Each guideline was rated by two researchers. When a difference in two or more points in each item was found, the item was discussed to get to a consensus. Otherwise, we used the mean of the two raters for each item. Lastly, we followed the AGREE-II Instrument guideline to calculate the scores for each domain [15].

We considered that when a CPG had a total score ≥ 70% it had adequate quality, we also used the same cutoff for each of the domains of the AGREE-II Instrument. This cutoff point was taken from a previous study that evaluated the quality of depression CPGs in children [8]. Likewise, we considered that when a CPG had a score ≥ 70% in the third domain (rigor of development) of the AGREE-II Instrument, the CPG had an adequate rigor of development.

## Results

We found twenty CPG for depression in adults published or updated between 2014 and 2018, of which thirteen performed systematic reviews to formulate their recommendations. Two CPGs were excluded from our study given that they were not available in full-text [16, 17], (See Additional file 2). Thus, we finally included eleven guidelines [18–28].

From the included guidelines, 2/11 included patients in the process of development of the CPG (one as part of the guideline development group [NICE], and 1/11 during the external validation [GuiaSalud]). Regarding how the development group reached the recommendations, 3/11 did not clearly state how recommendations were reached (Korea, RANZCP, USTF), 4/11 used expert consensus but did not specify the criteria evaluated (ACP, APA, VADoD, BAP), and 4/11 used a well-specified methodology (either: Grades of Recommendation, Assessment, Development, and Evaluation [GRADE], Scottish Intercollegiate Guidelines Network [SIGN], or Canadian Network for Mood and Anxiety Treatments [CANMAT]). All included guidelines specified the system they used for grading the strength of recommendations (Table 1).

**Table 1:** Characteristics of the included CPGs

Authors	Abreviation	Year	Country	Involvement of patients or their representatives	Methodology used to reach recommendations	Methodology used for grading the strength of recommendations	Use of minimally important difference
Canadian Network for Mood and Anxiety Treatments (CANMAT)	CANMAT	2016	Canada	No	CANMAT methodology	CANMAT	No
Depression Guideline Development Group (Acupuncture CPG)	Acupuncture CPG	2015	China	No	GRADE methodology	GRADE	No
National Collaborating Centre for Mental Health, National Institute for Health and Clinical Excellence (NICE)	NICE	2018	United Kingdom	Part of the guideline development group. Does not explicitly state what they did.	GRADE methodology	GRADE	Yes, they discuss the clinical importance of the effects, but a cut-off point is not clearly stated
Agencia de Evaluación de Tecnologías Sanitarias de Galicia (GuiaSalud)	GuiaSalud	2014	Spain	External validation of guideline	SIGN methodology	SIGN	Yes, they discuss the clinical importance of the effects, but a cut-off point is not clearly stated
American College of Physicians (ACP)	ACP	2016	United States of America	No	Expert consensus	ACP’s GRADE modification	Yes, they discuss the clinical importance of the effects, but a cut-off point is not clearly stated
American Psychiatric Association (APA)	APA	2010	United States of America	No	Expert consensus	APA (appears to be an expert consensus=	No
Royal Australian and New Zealand College of Psychiatrists	RANZCP	2018	Australia New Zealand	No	Not clear	Adaptation of NHMRC	No
US Preventive Services Task Force Recommendation Statement (US-Task Force)	USTF	2016	United States of America	No	Not clear.	USPSTF	Yes, they discuss the clinical importance of the effects, cut-off points are stated (eg. PHQ-9)
Management of Major Depressive Disorder Working Group (VA/DoD)	VADoD	2016	United States of America	No	Expert consensus	GRADE	No
Korean Guideline Development Team for Depression	Korea	2014	Republic of Korea	No	Not clear	SIGN plus their modifications	No
British Association for Psychopharmacology (BAP)	BAP	2015	United Kingdom	No	Expert consensus	BAP criteria	Yes. They state the cut-off points for each effect size measure.

The number of recommendations stated by each CPG varied between one and 199 recommendations. Three CPGs focused on one topic: the Acupuncture CPG [19] that aimed to give recommendations regarding acupuncture treatment, the ACP guideline [22] that aimed to determine the usage of pharmacological versus non-pharmacological treatment, and the US-Taskforce guideline [25] that aimed to state how the screening of depression should be performed. The other eight CPGs addressed multiple topics: one on non-pharmacological treatment (Korea), two on treatment (APA, RANZCP) [23, 24] and five on diagnosis and treatment (CANMAT, NICE, GuiaSalud, VADoD, BAP). Of note, three CPGs issued consensus statements (either GCPs or consensus-based recommendations).

In general, 6/11 CPGs provided recommendations on screening (CANMAT, NICE, GuiaSalud, USTF, VADoD, BAP), 5/11 on diagnosis (CANMAT, NICE, GuiaSalud, VADoD, BAP), 8/11 on pharmacological treatment (of which, six established the first line of treatment [CANMAT, GuiaSalud, APA, RANZCP, VADoD, BAP], and five established first and the second line of treatment [CANMAT, GuiaSalud, APA, RANZCP, BAP]), 9/11 on non-pharmacological treatments (all of them discussed psychological treatment [CANMAT, NICE, GuiaSalud, ACP, APA, RANZCP, VADoD, Korea, BAP], seven provided recommendations on the use of electro-stimulation therapy [CANMAT, NICE, GuiaSalud, APA, RANZCP, VADoD, BAP], six on St. John’s wort [CANMAT, NICE, GuiaSalud, APA, VADoD, BAP], and two on acupuncture [CANMAT, Acupuncture CPG]) (Table 2).

**Table 2:** Topics and number of statements issued by each CPG

Theme	CANMAT	WHO	NICE	GuiaSalud	ACP	APA	RANZCP	USTF	VADoD	Korea	BAP
Screening	1 R		1 R	4R 1 GCP				1 R	1 R		3 R 1 GCP
Diagnosis	1 R		4 R	1 GCP					1 R		1 R 3 GCP
Pharmacological treatment											
Pharmacological treatment: general	43 R		35 R^b^	8 R 5 GCP	1 R^a^	60 R^c^	5 R 9 CBR^a^		6 R		115 R 15 GCP
Pharmacological treatment: 1st line	1 R			3 R		6 R	3 R		1 R		7 R
Pharmacological treatment: 2nd line	1 R			2 R		3 R	1 R				7 R
Psychological treatment											
Psychological treatment: general	6 R		7 R^b^	2 R 1 GCP	1 R^a^	21 R^c^	3 R 3 CBR^a^		5 R		7 R 3 GCP
Psychological treatment: recommends at least one specific therapy	14R		15 R^b^	4 R		9 R	3 R		6 R	5 R	15 R
Other non-pharmacological treatments											
Electro-stimulation	9 R		11 R	4 R 2 GCP		16 R	7 R 2 CBR		5 R		20 R 6 GCP
Acupuncture	1 R	12 R									
St. John’s wort	1 R		1R	1 R 1 GCP		3 R			1 R		2 R
Light therapy	1 R					1 R			1 R		6 R
Exercise	1 R		1 R	2 R					1 R	1 R	3 R
Yoga	1 R										
Sleep deprivation	1 R										
Others treatments	10 R					3 R					4 R
Other statements	18 R		35 R	13 R 3 GCP		89 R	3 R 8 CBR		6 R	1 R	9 R 22 GCP
Total of statements	110 R	12 R	108 R	43 R 14 GCP	1 R^a^	211 R^c^	25 R 32 CBR	1 R	31 R	7 R	199 R 50 GCP

R: recommendations, GCP: good clinical practice, CBR: consensus-based recommendation

^a^ 01 recommendation included both pharmacological and psychological themes

^b^ 02 recommendations included both pharmacological and psychological themes

^c^ 06 recommendations included both pharmacological and psychological themes

To assess the quality of guidelines, we used the AGREE II instrument. The overall assessment score ranged from 53 to 87% (mean: 70.8%), and 4/11 guidelines had an overall assessment score ≥ 70% (NICE, ACP, USTF, VADoD). Regarding the third domain, scores ranged from 45 to 88% (mean: 62.9%), and 3/11 guidelines had a score ≥ 70% (NICE, ACP, VADoD) (Table 3).

**Table 3:** Quality appraisal of CPGs for depression in adults, using the AGREE-II instrument

CPG (code)	AGREE-II domains								Quality of the systematic reviews (Yes or No)			
	1	2	3	4	5	6	7	Overall assessment	Mentions databases used	Mentions search strategy	List of included studies is available	Mentioned risk of bias assessment as part of their methodology
Canadian Network for Mood and Anxiety Treatments (CANMAT)	92%	67%	53%	83%	0%	100%	67%	66%	Yes	Yes	No	No
Depression Guideline Development Group (WHO)	100%	67%	48%	89%	38%	8%	50%	57%	Yes	No	No	No
National Collaborating Centre for Mental Health, National Institute for Health and Clinical Excellence (NICE)	100%	100%	**85%**	100%	40%	100%	**83%**	87%	Yes	Yes	Yes	Yes
Agencia de Evaluación de Tecnologías Sanitarias de Galicia (GuiaSalud)	100%	92%	60%	94%	96%	100%	67%	87%	Yes	No	Yes	Yes
American College of Physicians (ACP)	100%	89%	**88%**	72%	0%	100%	**83%**	76%	Yes	Yes	Yes	Yes
American Psychiatric Association (APA)	94%	64%	55%	89%	15%	100%	67%	67%	Yes	No	No	No
Royal Australian and New Zealand College of Psychiatrists (RANZCP)	100%	81%	59%	100%	8%	100%	67%	74%	Yes	No	No	No
US Preventive Services Task Force Recommendation Statement (USTF)	94%	53%	65%	86%	17%	100%	**83%**	71%	Yes	Yes	Yes	Yes
Management of Major Depressive Disorder Working Group (VADoD)	94%	86%	**78%**	100%	17%	100%	**83%**	80%	Yes	Yes	No	Yes
Korean Guideline Development Team for Depression (Korea)	75%	47%	45%	78%	4%	83%	42%	53%	Yes	No	No	No
British Association for Psychopharmacology (BAP)	83%	56%	55%	78%	13%	83%	58%	61%	Yes	No	No	Yes

Find in boldface values above 70% which represents adequate quality

Regarding the quality of the systematic review, 11/11 of the included CPGs stated what databases they used, 5/11 CPGs published their search strategy (CANMAT, NICE, ACP, USTF, VADoD), 4/11 CPGs presented the included studies (NICE, GuiaSalud, ACP, USTF), and 6/11 CPGs mentioned the risk of bias assessment as part of their methodology (NICE, GuiaSalud, ACP, USTF, VADoD, BAP) (Table 3).

## Discussion

### Main findings

This study explores the characteristics, scope, and quality of CPGs for depression in adults that based their recommendations on systematic reviews and were published between January 2014 and May 2018. We included eleven CPGs from seven countries on four continents, from which two reported the patient involvement in the design or validation of the CPGs, six provided recommendations on screening, five on diagnosis, eight on pharmacological treatment, nine on psychological treatment, nine on other non-pharmacological treatments. Regarding the quality assessment, 4/11 CPGs reached a score ≥ 70% in the overall assessment of the AGREE-II instrument, and 3/11 CPGs reached a score ≥ 70% in the rigor of development domain. In addition, only 5/11 CPGs shared their search strategy, while only 4/11 listed the selected studies they used to reach recommendations, and 7/11 CPGs did not clearly state which methodology they used to translate evidence into a recommendation.

### Patient involvement

The involvement of patients or their representatives in the development of CPGs is considered important as it is supposed to complement scientific evidence to reach more acceptable and implementable recommendations [29]. Thus, many guidelines development groups recommend its inclusion in every step through the development of CPGs, including the definition of the scope and objectives, the definition of the review questions, the developing of recommendations (sharing their preferences regarding the assessed interventions), and the review of the final version of the CPG [30].

However, we found that 2/11 CPGs reported that patients had participated in the design or validation of the CPGs. This low patient involvement is similar to that found in other studies. One study that evaluated 62 Dutch guidelines assessed patients’ participation in the development process through three items (patients’ participation, identification of the patient’s input in the CPG, and the emphasis of patients’ participation in the individual patient level), and found that only 1/62 CPGs fulfilled satisfactorily these items [31]. The CPG that fulfilled these criteria was the Dutch guideline for depression [32]. Moreover, a study evaluated the patient involvement in guidelines in 101 organizations that publish CPGs in G-I-N North America and National Guideline Clearinghouse and found that only 8% of them require the patient or public involvement on guideline development groups, while 15% sometimes require it or describe it as optional [33].

### Quality of the CPGs

When focusing on the third domain of the AGREE II Instrument (rigor of development), few CPGs (3/11 = 27%) reached a score ≥ 70%. This is similar to a systematic review of CPGs for depression in children and youth that found that only 4/17 = 17.6% achieved a score ≥ 70% in this domain [8]. Other studies have reached different results regarding the percentage of CPGs that achieved a score ≥ 70% in the third domain on the AGREE-II instrument. i.e.: 1/11 = 9.1% for Heparin-induced thrombocytopenia CPGs [34], 15/115 = 13.0% for kidney transplantation CPGs [35], 5/30 = 16.6% for fertility preservation in young women undergoing gonadotoxic treatment CPGs [36], 6/17 = 35.3% for pediatric traumatic brain injury CPGs [37], and 5/12 = 41.7% for treatments for oral cancer CPGs [38].

Our results indicate that few of the CPGs achieve an adequate methodological quality, which could lead to recommendations that are not based on the best available evidence. This situation could be due to the fact that developing a high-quality CPG demands many financial resources, time, highly specialized personnel, and health system support [39–41]. In addition, some CPGs may fulfill an adequate rigor of development but attained a low score in the AGREE-II instrument because the development process was not adequately reported [42]. To avoid this, the guideline development groups could apply AGREE-II or another instrument to verify the adequate reporting of their CPGs.

To state a recommendation, two basic steps are needed: the selection of evidence and the methodology used to translate evidence into a recommendation [5]. We evaluated some characteristics in order to understand how these steps were performed.

Regarding the selection of evidence, only 5/11 CPGs shared their search strategy, while only 4/11 listed the selected studies they used to reach recommendations. Not sharing these information prevents the readers from adequately evaluating if there was any bias in the selection of evidence used to guide the recommendations, and prevents the replication and corroboration of the searches performed.

Regarding the methodology used to translate evidence into a recommendation, 7/11 CPGs did not clearly state the methodology used. A clearly defined methodology is necessary to understand what criteria were used and how the developing group judged each criterion to reach a recommendation. This allows users to understand how subjectivity and possible competing interest of the guideline developing group may have influenced on its recommendations, and help decide if recommendations can or should be implemented in their own settings [43]. Inconsistent recommendations are not rare, as shown by a systematic review that assessed the recommendations stated in CPGs for depression treatment, which found inconsistencies in the recommendations for the second and third line of pharmacological treatment [7]. For CPGs with inadequate methodology, it is necessary to evaluate the suitability of its use, and be careful when considering the implementation of its recommendations.

### Limitations and strengths

Our study is not free from limitations. We only collected guidelines published in English or Spanish, so our findings could not be representative of CPGs published in other languages. The CGPs quality was assessed using the AGREE-II instrument, based on the reporting of the CPGs, so guidelines with inadequate reporting could be classified as deficient, despite their actual quality. Lastly, there are not validated cut-off points for the AGREE-II instrument so the discrimination between CPGs with adequate and inadequate quality could be inaccurate.

However, to our knowledge, this is the first study that has evaluated the characteristics and quality of CPGs for depression in adults. This evaluation has some important strengths: we used a systematic search strategy involving eighteen databases to find available CPGs for depression in adults, we used the AGREE-II instrument that provides a standard methodology to critically appraise the quality of CPGs, and we performed independent appraisals by two researchers.

## Conclusions

We found eleven CPGs for depression in adults that used systematic reviews to guide their recommendations. Only two CGPs reported patient involvement. Regarding the quality of these CPGs, only 4/11 CPGs reached a score ≥ 70% in the overall assessment of the AGREE-II instrument, and 3/11 CPGs reached a score ≥ 70% in the rigor of development domain. In addition, only 5/11 CPGs shared their search strategy, while only 4/11 listed the selected studies they used to reach recommendations, and 7/11 CPGs did not clearly state which methodology they used to translate evidence into a recommendation. These findings should be taken into account by stakeholders, CPGs developers, and CPGs users; when choosing CPGs, and interpreting and putting into practice their issued recommendations from CPGs for depression in adults.

## Additional files

Additional file 1: Search strategies and results for CPGs for depression. We present the list of databases or CPG repositories, the search strategy and the results from the searches. (DOCX 14 kb)

Additional file 2: Flowchart of selection of CPGs. We present the flowchart of how we selected the CPGs that were included in our study. (DOCX 21 kb)

## Abbreviations

AGREE-II: Appraisal of Guidelines Research and Evaluation II
CANMAT: Canadian Network for Mood and Anxiety Treatments
CPGs: Clinical practice guidelines
GCP: Good clinical practice
GRADE: Grades of Recommendation, Assessment, Development, and Evaluation
MID: Minimally important difference
SIGN: Scottish Intercollegiate Guidelines Network
