# Supplementary material for: Characteristics and quality of clinical practice guidelines for depression in adults: a scoping review
Source: BMC Psychiatry. 2019 Feb 20;19:76. doi: 10.1186/s12888-019-2057-z (PMC6381686; doi:10.1186/s12888-019-2057-z)
Supplement: Supplementary file 1 — Search strategies and results for CPGs for depression. We present the list of databases or CPG repositories, the search strategy and the results from the searches. (DOCX 14 kb) [file 12888_2019_2057_MOESM1_ESM.docx]

## Supplementary material 1. Search strategies and results for CPGs for depression

| **Database** | **Link** | **Search terms and filters used** | **Total results** | **CPGs for depression in adults** |
| --- | --- | --- | --- | --- |
| Trip database | https://www.tripdatabase.com | Depression  Filter: guidelines | 619 | 12 |
| Canadian Medical Association Infobase: Clinical Practice Guidelines (CPG) | https://www.cma.ca/En/Pages/clinical-practice-guidelines.aspx | depression | 52 | 7 |
| eGuidelines | [www.eguidelines.co.uk](http://www.eguidelines.co.uk/) | depression | 49 | 1 |
| Guidelines International Network (G-I-N) | http://www.g-i-n.net/ | depression | 52 | 0 |
| National Guideline Clearinghouse (NGC) | https://www.guideline.gov/ | depression  Filter: target population adults (19 to 44 years) | 122 | 6 |
| National Health and Medical Research Council (NHMRC): Clinical Practice Guidelines | https://www.nhmrc.gov.au/guidelines-publications | depression | 4 | 0 |
| National Institute for Health and Care Excellence - UK (NICE) | https://www.nice.org.uk/ | depression  Filter: clinical guidelines | 77 | 1 |
| Scottish Intercollegate Guidelines Network (SIGN) | http://www.sign.ac.uk/ | Within current guidelines | 155 | 0 |
| Standards and Guidelines Evidence (SAGE) | http://www.cancerview.ca/TreatmentAndSupport/GRCMain/GRCSAGE/GRCSAGESearch/ | depression | 9 | 0 |
| American College of Physicians Clinical Practice Guidelines | https://www.acponline.org/clinical-information/guidelines | depression | 2 | 2 |
| Best Practice Guidelines | http://rnao.ca/bpg | depression  Filter by topic: depression | 3 | 2 |
| New Zealand Guidelines Group | http://www.health.govt.nz/about-ministry/ministry-health-websites/new-zealand-guidelines-group | depression  Filter: publication type – guides and standards | 3 | 0 |
| [CENETEC – Centro Nacional de Excelencia Tecnológica en Salud – Mexico](http://www.cenetec.salud.gob.mx/) | https://www.gob.mx/salud/cenetec | depresión | 0 | 0 |
| GuíaSalud. Guías de Práctica Clínica en el Sistema Nacional de Salud de España | http://portal.guiasalud.es | depresión | 9 | 2 |
| IETS Colombia | http://www.iets.org.co/ | Within all published guidelines | 26 | 1 |
| Medline | https://www.ncbi.nlm.nih.gov/pubmed | (Depression[TI] OR "Depressive Disorder"[Mesh] OR "Depression"[Mesh]) AND  (guideline[Publication Type] OR guideline*[TI] OR recommendation*[TI])  Filter: published within the last 5 years | 256 | 36 |
| Epistemonikos GRADE guidelines repository | https://www.epistemonikos.org/en/ | Depression  Filter by category broad syntheses | 11 | 0 |
| Google | http://google.com | depression guideline recommendation  We reviewed the firsts 100 hits | 100 | 13 |

*Note:* Last search was made on June 2018.
