# Supplementary material for: Characteristics and quality of clinical practice guidelines for depression in adults: a scoping review
Source: BMC Psychiatry. 2019 Feb 20;19:76. doi: 10.1186/s12888-019-2057-z (PMC6381686; doi:10.1186/s12888-019-2057-z)
Supplement: Supplementary file 2 — Flowchart of selection of CPGs. We present the flowchart of how we selected the CPGs that were included in our study. (DOCX 21 kb) [file 12888_2019_2057_MOESM2_ESM.docx]

## Supplementary material 2. Flowchart of search strategy and clinical practice guideline selection

Records identified through database searching
(n = 1549)

Records excluded
(n = 1475)

Full-text articles assessed for eligibility
(n = 74)

Full-text articles excluded (n = 57):

- Not CPG (n=9)
- Not adults/general population (n=39)
- Full-text unavailable (n=2)
- Do not perform systematic reviews (n=7)

17 Included records

(11 Sets of guidelines)
